# Cold Atmospheric Plasma Treatment of Zirconia to Improve Its Bond Strength and Longevity with Dental Cement

**DOI:** 10.3390/ma18153482

**Published:** 2025-07-24

**Authors:** Yixuan Liao, ThiThuHa Phan, Qingsong Yu

**Affiliations:** 1 Department of Mechanical and Aerospace Engineering, University of Missouri, Columbia, MO 65211, USA; 2 Department of Chemical and Biomedical Engineering, University of Missouri, Columbia, MO 65211, USA

**Keywords:** zirconia, argon cold atmospheric plasma, micro-shear bond strength, dental cement, bond longevity

## Abstract

Zirconia restoration debonding is one of the common issues in its dental applications because of its dense and chemically inert structure that is difficult to bond to. In this study, plasma treatment of zirconia was performed to improve its bond strength and longevity with dental resin cement. Sandblasted zirconia specimens were treated using argon cold atmospheric plasmas (CAPs), followed by applying a thin layer of 10-MDP primer, dental resin cement with light curing. Micro-shear bond strength (µSBS) test results showed that 300 s of CAP treatment significantly increased the initial µSBS to 38.3 ± 5.6 MPa as compared with the 21.6 ± 7.9 MPa without CAP treatment. After 30 days of storage in 37 °C deionized (DI) water, CAP-treated zirconia specimens had 191.2% higher bond strength than the bonded specimens without plasma treatment. After 1000 cycles of thermal cycling (TC) between 5 °C and 55 °C, the CAP-treated zirconia specimens gave 30.5% higher bond strength than the bonded specimens without plasma treatment. Surface–water contact angle measurements indicated that the zirconia surface became much more hydrophilic but showed rapid hydrophobic recovery within the first hour of CAP treatment, indicating the importance of promptly applying the primer after the plasma treatment. These findings suggest that the argon CAP technique is effective in the surface preparation of zirconia for enhancing bond strength and longevity with dental cement.

## 1. Introduction

In recent years, there has been a growing demand for all-ceramic dental restorations, leading to the development of ceramic materials with optimized mechanical properties, such as dense-sintered alumina and zirconia ceramics [1,2]. These high-strength ceramics have found extensive use in various clinical applications, including pillars, fixed partial dentures, and implant abutments. While an adhesive bond is recommended for all-ceramic dental restorations [3], ensuring a long-term and durable bond is crucial, particularly for applications such as crowns, veneers, and implant abutments.

Zirconia (zirconium dioxide, ZrO_2_), one type of restorative ceramic material, possesses favorable properties of high flexural strength, high thermal conductivity, excellent esthetics, and superior fracture resistance [4,5]. This type of ceramic material can be utilized in various dental positions, including crowns, bridges, veneers, posterior three-unit fixed partial dentures, endodontic dowels, and implant abutments. Despite the desirable properties of zirconia for dental applications, conventional ceramic surface modification methods such as hydrofluoric acid etching and silane application are not effective for achieving a reliable bond to zirconia due to its silica-free nature [6]. Therefore, it is essential to identify an appropriate surface modification method that can ensure reliable and durable adhesion to the chemically inert zirconia surface, thereby ensuring long-term bond reliability between zirconia and dental resin cement [7]. The various surface modification methods that have been explored to enhance the bond strength between zirconia and resin cement include airborne particle abrasion with aluminum oxide, tribochemical silica coating, selective infiltration etching, fluorination vapor technique, and laser irradiation [8,9]. Zirconia restorations, however, often show unsatisfactory bond durability due to their chemically inert surface, resulting in significant bond strength degradation after thermocycling and aging [10]. It has been reported that many adhesive systems fail to maintain stable long-term adhesion to zirconia, with frequent loss of retention observed under in vitro conditions simulating clinical aging [11,12]. Therefore, there is still a need to further explore new technologies for the surface preparation of zirconia to improve its adhesion properties and long-term bond stability.

One promising technology to improve the adhesion properties of zirconia is cold atmospheric plasma (CAP) treatment. CAP is a partially ionized gas or low-temperature plasma that is generated at atmospheric pressure and suitable for clinical applications in dentistry [13]. In CAP, there are many reactive plasma species including electrons, ions, free radicals, and electronically excited atoms/molecules, etc. Such technology has been found to be very effective in oral bacterial inactivation [14,15] and bond strength enhancement in dental restoration applications [16,17,18]. It was reported that argon CAP treatment on dentin surfaces significantly enhances composite-to-dentin bond strength, with studies showing up to a 64% increase in micro-tensile bond strength after just 30 s of plasma exposure [19,20].

Recently, CAP treatment of zirconia has been actively studied to improve its bond with resin cements for restorative applications. Ito et al. [21] reported that CAP treatment of zirconia enables the removal of carbon derived from organic contaminants by using X-ray photoelectron spectroscopy (XPS) analysis of the samples. It was also reported that 5 min argon CAP treatment of zirconia increased its micro-shear bond strength with resin cement by 88.6% as compared with the untreated sample [22]. Studies by Ye et al. [23] and Muira et al. [24] demonstrated that helium and argon CAP treatment significantly increased the shear bond strength of zirconia with resin cement without compromising surface integrity. In these reported studies, however, initial bond strength was mostly evaluated after storing the bonded zirconia samples in deionized water or humid air at 37 °C for 24 h. As pointed out by Rigos et al. [12], studies evaluating the durability of bond strength are the most appropriate when determining the most successful protocol for clinical use. This study, therefore, aims to investigate the surface treatment of zirconia by CAP to improve its bond durability and longevity with dental resin cement. The hypothesis is that CAP treatment can activate the zirconia surface to better bond with dental resin cement for improved micro-shear bond strength longevity.

## 2. Materials and Methods

### 2.1. Zirconia Test Specimen Preparation

Zirconia plates with a dimension of 5.0 mm × 5.0 mm × 10.0 mm were obtained from a 94% ZrO_2_ stabilized by 5% Y_2_O_3_ ceramic (Cercon Smart Ceramics, Degudent, Hanau, Germany). They were sintered at 1500 °C in a sintering furnace. For test specimen preparation, these zirconia plates were first embedded in acrylic resin (Allied, CA, USA) using cylindrical embedding molds. The acrylic resin was prepared by mixing QuickCure Acrylic Powder (Allied, Cerritos, CA, USA) and QuickCure Acrylic Liquid (Allied, Cerritos, CA, USA) at the specified ratio of two parts of the powder with one part of the liquid. In the safety data sheet for QuickCure, the term “Acrylic Liquid” refers to mixture of chemical compound methyl methacrylate and N,N-dimethyl-p-toluidine, while “Acrylic Powder” pertains to Poly(methyl methacrylate) (PMMA, CAS number 9011-14-7). The prepared acrylic resin solution was added into an embedding mold with a zirconia sample placed at the bottom surface center position. The resin-filled mold was then placed in an ice-water bath to cool for 1 h. This step helps uniform polymerization and curing of the resin and ensures a proper embedding of the zirconia specimens. Once the resin was fully polymerized and cured, the embedded zirconia resin specimens were carefully extracted from the mold. The polymerized resin, along with the embedded zirconia with an exposed surface area of 5.0 mm × 5.0 mm, was then stored in 4 °C deionized (DI) water until use.

**Figure 1:**
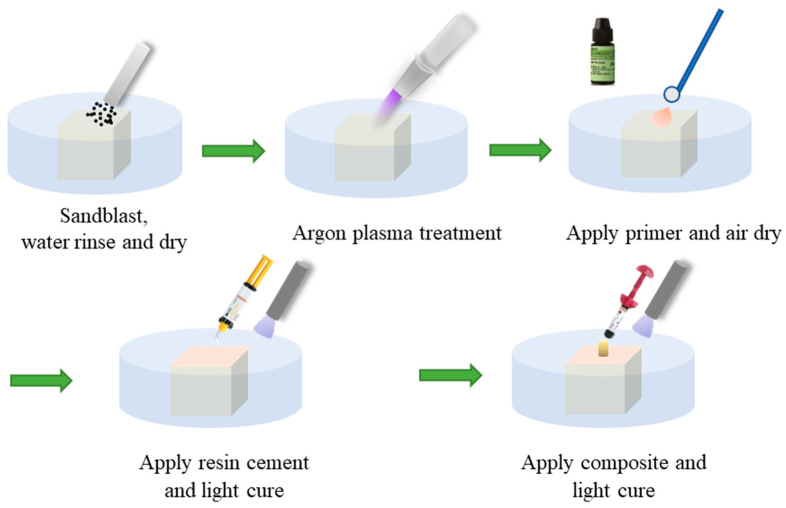
Schematic of the zirconia (zirconium oxide, ZrO_2_) test specimen preparation procedures.

The test specimen preparation and bond procedures for the zirconia with dental cement are illustrated in Figure 1. The exposed surface of the zirconia embedded in the resin was first sanded flat by a Trimmer machine (JT19, Zeny, Fontana, CA, USA) and then polished using 600-grit polish papers (Norton Black Ice T401 9” × 11” Wet/Dry Silicon Carbide Sheets, 600 Grit, Norton, Worcester, MA, USA). Then the specimen surfaces were sandblasted using 0.3 µm Al_2_O_3_ particles applied perpendicular to the surface at 0.28 MPa for 20 s at a distance of about 10 mm. The sandblasted zirconia surface was ultrasonically cleaned in 99% isopropyl alcohol (Sigma-Aldrich, St. Louis, MO, USA) for 5 min and then air-dried. With (plasma-treated groups) or without (untreated control group) going through plasma treatment, the zirconia surface was primed with a very thin layer of primer (Z-prime™ plus, Bisco, Inc., Schaumburg, IL, USA) and gently dried with compressed air. In this study, Z-Prime™ Plus was selected because it reportedly provided the highest shear bond strength to zirconia among four different primers with 3 resin cements including RelyX™ Unicem [25]. Subsequently, ~0.45 mm thick resin cement (RelyX™ Unicem 2 Automix Self-Adhesive Resin Cement, 3M, St. Paul, MN, USA) was applied to the primed zirconia surface and light cured using an LED dental light (Superdental LED light, North Andover, MA, USA) for 10 s. The chemical composition information on the ingredients of Z-prime™ plus primer is summarized in Table 1. The specimen was finally fixed on an Ultradent mold (Bonding Clamp and Bonding Mold Inserts, Ultradent Products Inc., South Jordan, UT, USA). A dental composite was put (Universal Restorative-A3 Shade, 3M, St. Paul, MN, USA) through the mold to form a composite post on top of the resin cement and it was light cured first for 10 s and then for another 10 s. Any excess flash material on the base of the composite post was carefully removed using a razor blade.

In the plasma-treated sample groups, argon CAP treatment was performed on the sandblasted zirconia surfaces for 30 s, 60 s, 90 s, 120 s, and 300 s. A total of 15 specimens were prepared for each of the specimen groups. All the specimens were first stored in 37 °C DI water for 24 h. Then, some of the specimens were subjected to the micro-shear bond strength (µSBS) test to evaluate the initial bond strength. The remaining specimens were divided into two groups, with one group being stored for an additional 30 days in 37 °C DI water and the other group undergoing thermal cycling to evaluate the bond durability and longevity. Thermal cycling was performed by alternatingly storing the specimens between two water baths with temperatures of 5 °C and 55 °C for 1000 cycles. All the specimens were handled and disposed of according to the protocols suggested by the Environmental Health and Safety Department at the University of Missouri, Columbia, MO, USA.

### 2.2. Cold Atmospheric Plasma Treatment

CAP treatment of the zirconia was performed using a lab-made plasma brush device, with the information detailed in our previous study [26]. Argon gas (ultra-high purity, Airgas, Radnor Township, PA, USA) was employed as the plasma operating gas with a flow rate of 3000 standard cubic centimeters per minute (sccm). The CAP plasma jet blown out from a ceramic by argon gas flow has a brush shape with a gas-phase temperature of <40 °C. The argon CAP was operated at a current of 10 mA and voltage of 1.20 kV using a high-voltage DC power supply (SL60, Spellman, New York, NY, USA).

### 2.3. Micro-Shear Bond Strength (μSBS) Testing

The μSBS tests were performed using Ultradent’s micro-shear bond strength (µSBS) test method [27] with a notched-edge crosshead mounted to an Instron universal testing machine (Instron 3367 Dual Column Testing Systems, Instron, Norwood, MA, USA). To perform the test, a bonded zirconia test specimen was placed in an Ultradent metal sample holder. The holder was then placed under the testing notched-edge crosshead by aligning the notched-edge centered over the composite button of the test specimen. During the test, a shear force was applied to the small, bonded area of a test specimen with a crosshead speed of 1 mm/min till the specimen fractures. The maximum force was recorded for each test specimen.

### 2.4. Surface–Water Contact Angle Measurements

The zirconia surface was characterized using water contact angle measurements that were performed using a computer-aided VCA 2500 XE Video Contact Angle System (AST Products, Inc., Billerica, MA, USA). After a 0.5 µL droplet of distilled water was placed onto the zirconia surface, optical images of the water droplet were recorded and analyzed using the software Image J version 1.53k (U.S. National Institutes of Health, Bethesda, MD, USA) to determine the water contact angles. To study the aging effect of the plasma treatment, water contact angles of the plasma-treated zirconia surfaces were measured with different time intervals of 30 s, 1 min, 2 min, 5 min, 10 min, 1 h, 2 h, 5 h, and 1 day after the argon CAP treatment.

### 2.5. Surface Examination

The zirconia surface was examined using an optical microscope (AmScope, Irvine, CA, USA) and an optical profilometer (Veeco NT 9109, Veeco Model, Plainview, NY, USA). The surface morphology and surface roughness data were acquired for the untreated zirconia, sandblasted zirconia, and 300 s plasma-treated zirconia.

### 2.6. Data Analysis

The μSBS test data was statistically analyzed using an ANOVA and Tukey’s test with OriginPro 2022 (OriginLab Corp, Northampton, MA, USA). The μSBS test data was then presented using the mean ± standard deviation (SD). The difference between test groups was considered to be statistically significant with *p* < 0.05 (* represents *p* < 0.05, ** represents *p* < 0.01, *** represents *p* < 0.001, **** represents *p* < 0.0001).

## 3. Results

### 3.1. Micro-Shear Bond Strength (μSBS) Test Results

**Figure 2:**
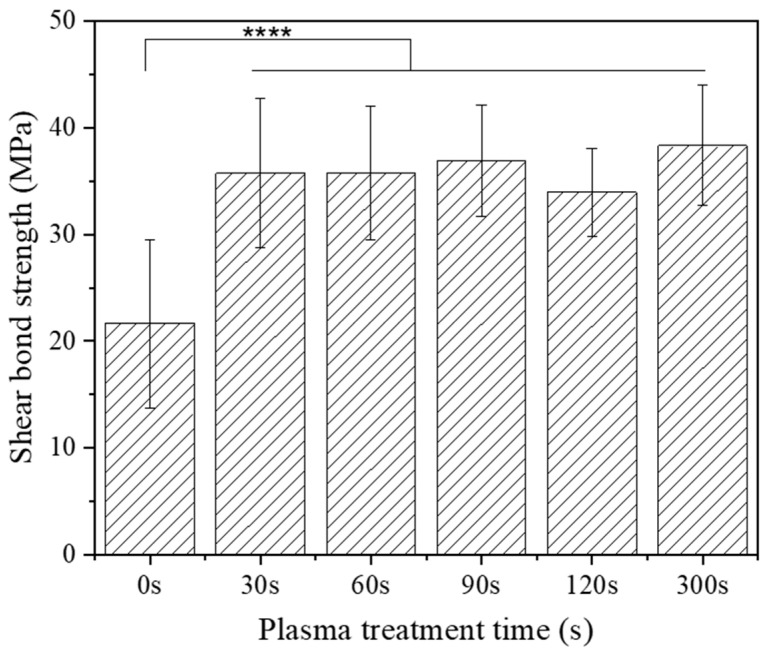
Initial micro-shear bond strength (µSBS) test results of zirconia-resin with different plasma treatment times using argon CAP. (****: *p* < 0.0001). The bonded zirconia specimens were stored in 37 °C DI water for 24 h before performing µSBS test.

As illustrated in Figure 2, the μSBS of the untreated zirconia control samples that underwent sandblasting alone was 21.6 ± 7.9 MPa. In contrast, CAP-treated zirconia sample groups gave significantly higher bond strengths of 35.7 ± 7.0 MPa, 35.8 ± 6.2 MPa, 36.9 ± 5.2 MPa, 34.0 ± 4.1 MPa, and 38.3 ± 5.6 MPa, with plasma treatment time of 30 s, 60 s, 90 s, 120 s, and 300 s, respectively. Notably, the zirconia–resin cement bond strength increased by 77.3% when the zirconia samples were treated with an argon plasma brush for 300 s compared to the untreated zirconia control samples. Statistical analysis confirmed a significant difference between the control group and the CAP-treated zirconia sample groups. However, there was no significant difference observed among the CAP-treated zirconia sample groups with different plasma treatment times.

**Figure 3:**
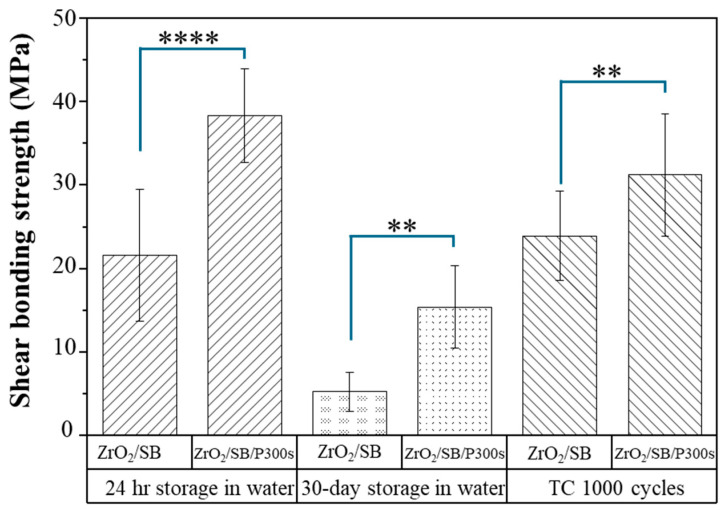
Comparison of micro-shear bond strength (µSBS) test results of 0 s or 300 s argon plasma-treated zirconia (zirconium oxide, ZrO_2_)–resin cement specimens that went through 1000 cycles of thermocycling. (**: *p* < 0.01; ****: *p* < 0.0001; ZrO_2_/SB: sandblasted zirconia; P300s: 300 s plasma treatment; 24 h storage in water: 24 h storage of the bonded samples in 37 °C deionized water; 30-day storage in water: 30 days of storage of the bonded samples in 37 °C deionized water; TC 1000 cycles: the bonded samples went through 1000 cycles thermal cycling).

Figure 3 illustrates the μSBS test results of zirconia test specimens that went through 30 days of storage in 37 °C deionized (DI) water, and those that went through 1000 cycles of thermal cycling between 5 °C and 55 °C to mimic an oral environment. The results indicated that the CAP-treated zirconia samples exhibit significantly higher bond strength than the control group with no CAP treatment. After 30 days of storage in 37 °C DI water, both the untreated zirconia sample group and 300 s CAP-treated zirconia sample group showed reduced bond strength compared with the test specimens after only 24 h storage in 37 °C DI water. However, the CAP-treated zirconia test specimens still maintained a significantly higher bond strength, which was 191.2% higher than the untreated zirconia sample group with no plasma treatment. For the zirconia test specimens that were subjected to 1000 cycles of thermal cycling, the CAP-treated zirconia sample group exhibited significantly higher bond strength compared with the untreated zirconia sample group with no plasma treatment. After 1000 cycles of thermal cycling, the bond strength of the CAP-treated zirconia sample group exhibited a slightly reduced bond strength compared with the test specimens that went through only 24 h storage in 37 °C DI water. It was noted that, however, the CAP-treated zirconia sample group still showed 30.5% higher bond strength than the untreated zirconia sample group with no plasma treatment. Overall, the results indicated that CAP treatment of zirconia improved the micro-shear bond strength between zirconia and resin cement and enhanced the durability of the interfacial bond, even after 30 days of storage in 37 °C DI water and 1000 cycles of thermal cycling, which mimics an oral environment.

### 3.2. Surface–Water Contact Angle Changes

**Figure 4:**
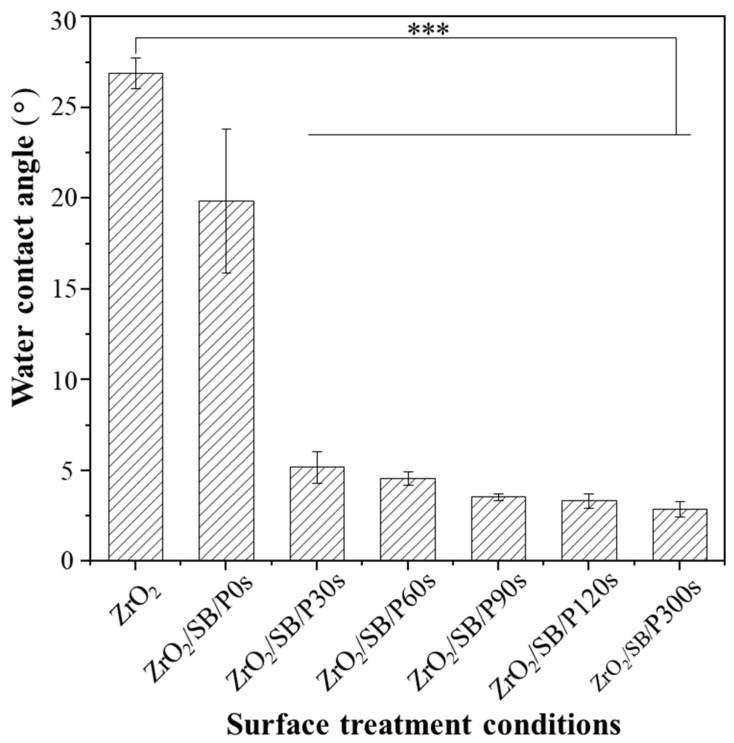
Change in water surface contact angle on zirconia (zirconium oxide, ZrO_2_) surface with different plasma treatment time. (***: *p* < 0.001; SB: sandblasted; P0s: 0 s plasma treatment = no plasma treatment; P30s: 30 s plasma treatment; P90s: 90 s plasma treatment; P120s: 120 s plasma treatment; P300s: 300 s plasma treatment).

The water contact angle changes in the zirconia surfaces were measured after sandblasting and argon CAP treatment for different durations. As seen in Figure 4, there is a slight decrease in the water contact angle of the zirconia surface after sandblasting. In contrast, CAP treatment on the sandblasted zirconia surface significantly reduced the water contact angle. This indicated that argon CAP treatment enhanced the surface hydrophilicity and wettability of zirconia. The longer the duration of CAP treatment, the lower the water contact angle on the zirconia surface. Among the CAP-treated zirconia sample groups, the zirconia samples treated with plasma for 300 s exhibited the lowest water contact angle, approaching ~ 3 degree, with a highly hydrophilic surface being achieved. Overall, the results demonstrated that argon CAP treatment of zirconia significantly improved its surface hydrophilicity and water wettability.

**Figure 5:**
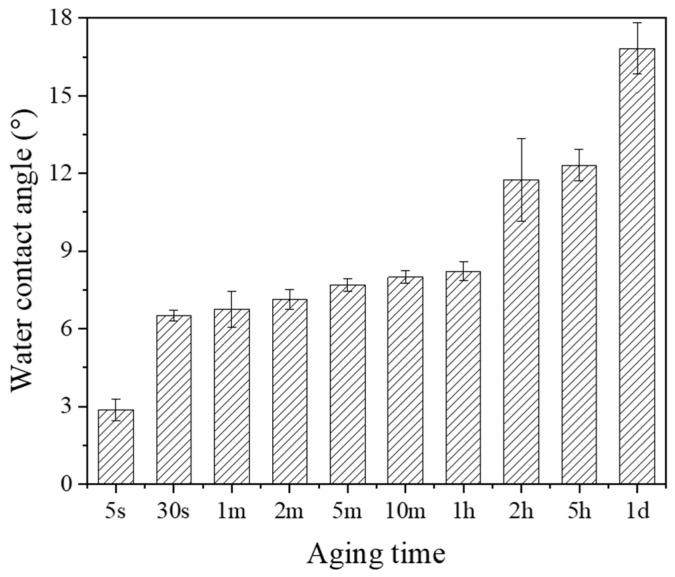
Water contact angle changes in plasma-treated zirconia (ZrO_2_/SB/P300s) surface with aging time. (SB: sandblasted, P300s: 300 s plasma treatment).

As illustrated in Figure 5, the aging time of a CAP-treated zirconia surface significantly affected the measured water contact angle. It was noted that the zirconia surface showed rapid hydrophobic recovery after CAP treatment. The water surface contact angle jumped up quickly within the first 30 s after the CAP treatment, and then gradually increased thereafter. After 1 day, the CAP-treated zirconia surfaces had a water contact angle similar to the sandblasted zirconia surfaces without CAP treatment. The results indicate that the surface hydrophilicity and water wettability of the CAP-treated zirconia kept reducing with aging time. In other words, the CAP treatment effect on the zirconia surface gradually diminished over time. Based on these findings, it is suggested that dental cement should be applied immediately after the CAP treatment to achieve a better bond improvement.

### 3.3. Surface Morphology and Surface Roughness Changes

**Figure 6:**
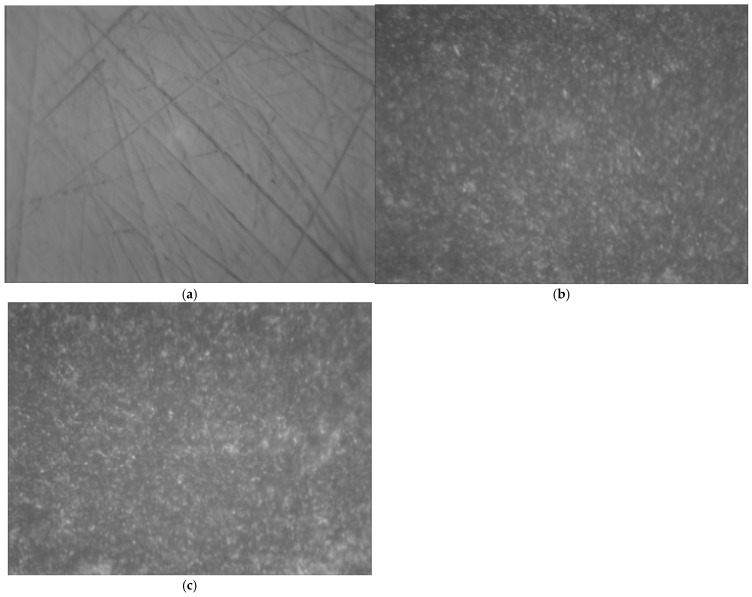
Optical microscopic images (100× magnification) of zirconia (zirconium oxide, ZrO_2_) surfaces with (**a**) no treatment; (**b**) SB: sandblasted; (**c**) SB/P300s: sandblasted and then plasma-treated for 300 s.

**Figure 7:**
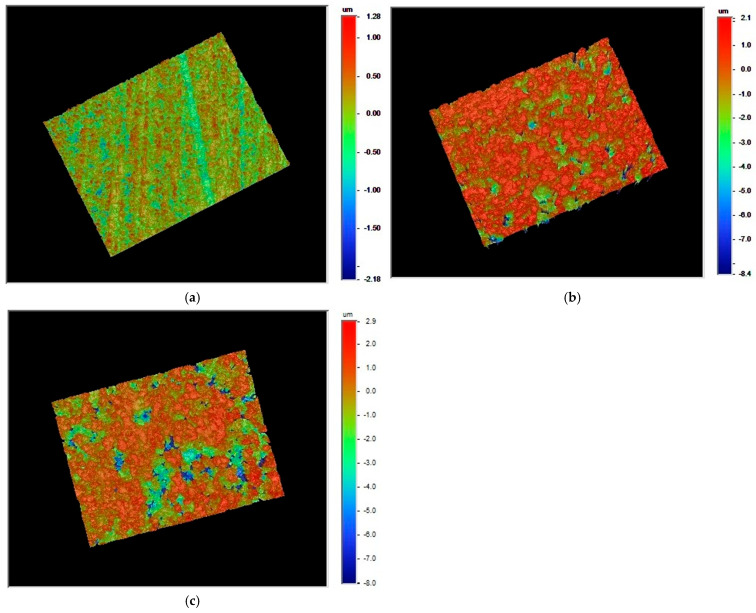
Surface morphology images of zirconia (zirconium oxide, ZrO_2_) surfaces with (**a**) no treatment; (**b**) SB: sandblasted; (**c**) SB/P300s: sandblasted and then argon plasma-treated for 300 s.

Figure 6 displays the optical microscopic images of zirconia surfaces before and after sandblasting, and after CAP treatment as well. As seen in Figure 6, the zirconia surface without any treatment (Figure 6a) exhibited obvious scratches caused by the 600-grit sandpaper, as observed under an optical microscope. After sandblasting (Figure 6b), however, the scratches on the zirconia surface became less visible, and the surface morphology became rougher due to the abrasive particles. After 300 s of CAP treatment on the sandblasted zirconia surface (Figure 7c), the surface morphology remained similar to that of the sandblasted zirconia (Figure 6b). In other words, the argon CAP treatment had little effect on the morphology of the zirconia surface.

Figure 7 and Table 2 illustrate the surface morphology analysis results of zirconia surfaces using an optical profilometer. It was noted that, in comparison with the untreated zirconia (Figure 7a), the roughness of the zirconia surface significantly increased after sandblasting (Figure 7b). In contrast, CAP treatment on the sandblasted zirconia surfaces had very little effect on the roughness of the zirconia surfaces. Considering the standard deviations, the roughness data in Table 2 do not demonstrate a definitive difference between zirconia surfaces that were sandblasted with or without an additional 300 s plasma treatment. As seen in Figure 7, the surface morphology images of sandblasted zirconia without CAP treatment (Figure 7b) and with CAP treatment (Figure 7c) are essentially indistinguishable. These results were consistent with the observations from the optical microscope images shown in Figure 6. These results indicate that sandblasting significantly increased the surface roughness of zirconia. However, subsequent argon CAP treatment had little effect on zirconia surface roughness.

## 4. Discussion

In the field of dentistry, achieving a stable and durable interface bond between zirconia and resin cement has been a longstanding challenge. Although various surface modification methods have been explored to improve the bond strength of zirconia, there is still no consensus on a standardized pretreatment method for zirconia surfaces [8,9,28]. As a novel technology for surface treatment and modification, CAP has demonstrated positive effects on the adhesion of dentin, enamel, and other dental materials in research studies [17,29,30]. In this study, CAP was used to treat zirconia to improve its bond strength with dental resin cement. Especially, the durability of the μSBS bond strength was evaluated. μSBS tests were used to evaluate the bond strength because zirconia mostly experiences shear stress for its commonly used dental applications, such as crowns or veneers. To assess the CAP treatment effects on zirconia surfaces, water contact angle measurements and surface roughness examination were performed. Water contact angle analysis provides insights into the surface free energy and hydrophilicity changes in the plasma-treated zirconia surfaces. The results from the previous section indicate that CAP treatment improved the μSBS bond strength and durability between zirconia and resin cement.

### 4.1. CAP Treatment Effects of Zirconia on Bond Strength and Durability

In this study, plasma treatment of the sandblasted zirconia surfaces significantly increased μSBS bond strength with resin cement compared to the sandblasted zirconia sample with no CAP treatment. As shown in Figure 2, CAP treatment resulted in an increase up to 77.3% in μSBS bond strength compared to the control group without plasma treatment. These results highlight the effectiveness of plasma treatment in improving the adhesion properties of zirconia. It was reported that air plasma treatment introduces hydrophilic functional groups onto inactive substances like zirconia and titanium, enhancing their adhesive properties [31,32]. The argon plasma treatment could improve the adhesion between dentin or enamel with adhesives by reducing surface hydrophilicity, i.e., increasing the surface energy of the substrate surface [17,29,30]. These results demonstrated that argon CAP treatment is effective for the surface modification of zirconia to enhance its bond strength with dental cement for various dental applications.

To evaluate the bond longevity, μSBS tests were also performed on zirconia test specimens after 30 days of storage in DI water and after thermocycling. As shown in Figure 3, the μSBS bond strength of all the test groups decreased after 30 days of storage in DI water. However, zirconia samples treated with a 300 s argon CAP exhibited significantly higher bond strength compared to the zirconia sample group with no CAP treatment. The results suggest that the CAP-treated zirconia specimens maintained their enhanced bond strength for a much longer period than the zirconia sample group with no CAP treatment. The thermocycling process simulates the long-term consumption of restorative materials in an oral environment. After 1000 cycles of thermal cycling, the bond strength of the 300 s argon CAP-treated group decreased to some extent. Despite the decrease, however, the CAP-treated zirconia specimens still exhibited significantly higher bond strengths than the zirconia sample group with no CAP treatment. In overall, the μSBS test results demonstrated that argon CAP treatment of zirconia is effective in enhancing its bond strength with dental resin cement. In addition, it also has a positive effect on the long-term stability of the bond as it helps maintain a higher bond strength even after aging processes such as prolonged storage and thermal cycling. These results suggest the great potential of CAP treatment in enhancing the bond durability of zirconia in dental applications.

### 4.2. CAP Treatment Effects on Surface Energy and Surface Morphology of Zirconia

The surface wettability is one of the important factors that may influence the bond strength of zirconia–resin cement. An improved surface wettability can maximize the contact of the primer to the zirconia surfaces and consequently enhance the bond of dental cement to zirconia substrates. After CAP treatment, as shown in Figure 4, the sandblasted zirconia surfaces had water contact angles decreased to ~3–5°. Such low water contact angles indicate a very hydrophilic zirconia surface with high surface energy, which will help improve the bond with dental cement.

Another important factor that affects the bond strength is the surface roughness of zirconia. As observed from Figure 6 and Figure 7 and Table 2, CAP treatment did not change surface roughness of the sandblasted zirconia. This indicates that plasma treatment did not alter the surface morphology of the zirconia but rather increases its surface hydrophilicity or water wettability. Similar observations have been reported in other studies on the plasma treatment of different materials [33,34,35]. In summary, argon CAP treatment did increase the water wettability and surface hydrophilicity of zirconia without significantly altering its surface morphology.

### 4.3. Plausible Mechanisms of CAP Treatment for Bond Enhancement

CAP contains various energetic and reactive plasma species, including energetic free electrons, ions, and electronically excited metastable atoms or molecules. In argon CAP, various reactive oxygen species (ROS) and nitrogen species (RNS), such as excited O atoms and electronically excited N_2_ molecules, are formed through plasma reactions in ambient air that contains mainly oxygen and nitrogen [36]. These plasma reactive species could inject more energy onto zirconia surfaces and consequently induce surface activation, which was reflected in the significant reduction in the water contact angle, i.e., the surface energy increase for the CAP-treated zirconia.

Resin cements are hydrophobic materials that do not directly bond to ceramic surfaces. To enhance the bond between resin cement and ceramics, various surface treatments and primers have been developed. Silane coupling agents, commonly used for silica-based ceramics, create a Si-OH group through a hydrolysis reaction and improve the bond strength between the ceramic and resin cement. However, the adhesion of silane coupling agents to zirconia ceramics, which lacks silica content, is not significantly improved due to their chemical characteristics. In the case of zirconia, primers containing 10-methacryloyloxydecyl dihydrogen phosphate (10-MDP) have been recommended to enhance bond strength. As shown in Table 1, the Z-primer used in this study contains 10-MDP. Compared to silane coupling agents, 10-MDP primers have functionalized phosphorus atoms that can attach to zirconium atoms, resulting in improved bond performance with zirconia [37]. The surface activation of zirconia by CAP treatment could further enhance such chemical reactions to form strong bonds with zirconia and thus significantly enhance bond strength and durability, as observed in this study.

In this study, argon CAP treatment of zirconia showed effective surface activation and significantly enhanced bond strength and durability with dental cement. CAP treatment of zirconia significantly increases its surface hydrophilicity but did not alter its surface morphology. This study, however, has some limitations. Only argon CAP with fixed plasma parameters except treatment time was used to treat the zirconia surfaces. Future research should include investigating the effects of different plasma operation parameters, such as electrical power inputs and plasma gas compositions, to optimize the application conditions of CAP. In addition, as postulated by Gale and Darvell [38], 10,000 thermal cycles approximately correspond to 1 year of clinical function. Long-term water storage and thermal cycling evaluation with >10,000 cycles are necessary to distinguish clinically durable zirconia bonding systems from non-durable zirconia bonding systems [12,38].

## 5. Conclusions

Zirconia is a popular dental restorative material due to its mechanical properties and esthetic appeal. However, its lack of silica content makes it difficult to establish a reliable bond with traditional methods such as etching with hydrofluoric acid. In this study, argon CAP treatment was used to activate zirconia surfaces to improve their bond strength and durability with dental cement. The main findings include the following:

- The CAP treatment significantly enhanced the initial bond strength between zirconia and resin cement, with micro-shear bond strength increased to 38.3 ± 5.6 MPa (a 77.3% increase) as compared with the 21.6 ± 7.9 MPa of the controls without plasma treatment.
- The plasma treatment led to a decrease in the water surface contact angle of the zirconia, resulting in increased water wettability, surface hydrophilicity, and surface energy without altering its surface morphology.
- After 30 days of storage in 37 °C deionized (DI) water and 1000 cycles of thermal cycling (TC) between 5 °C and 55 °C, more durable bond strength was observed with the CAP-treated zirconia specimens, highlighting the long-term stability of the interfacial bond achieved with CAP treatment.

In summary, CAP treatment is a promising approach for achieving a strong and durable bond between zirconia and dental cement, overcoming the challenges associated with the absence of silica content in zirconia that resulted in difficulties in achieving a strong bond with dental resin cement. Future research is required to investigate the effects of different plasma operation parameters and optimize the application conditions of CAP. Long-term water storage and thermal cycling evaluation with >10,000 cycles are necessary to determine the most successful protocol for clinical use.

## Figures and Tables

**Table 1:** Main chemical composition of Bisco Z-prime™ plus primer ^1^.

Components	%
Ethanol	75–85
Bisphenol A-glycidyl methacrylate (BisGMA)	5–10
2-hydroxyethyl methacrylate	5–10
10-methacryloyloxydecyl dihydrogen phosphate (10-MDP)	1–5
Triethylamine	<1

^1^ Z-prime™ plus safety data sheet.

**Table 2:** Surface roughness results of zirconia (zirconium oxide, ZrO_2_) samples.

Zirconia Samples	*Ra* (nm)	*Rq* (nm)
Untreated zirconia	282 ± 76	336 ± 61
Sandblasted zirconia	867 ± 64	1190 ± 17
Sandblasted and 300 s plasma-treated zirconia	924 ± 49	1227 ± 59

## Data Availability

The original contributions presented in this study are included in the article. Further inquiries can be directed to the corresponding author.

## Abbreviations

The following abbreviations are used in this manuscript:

CAPs	Cold atmospheric plasmas
DI	Deionized
µSBS	Micro-shear bond strength
RNS	Reactive nitrogen species
ROS	Reactive oxygen species
TC	Thermal cycling
